# Medical Practice in Palestine

**Published:** 1934

**Authors:** H. J. Orr-Ewing

**Affiliations:** Late Consultant Physician, Government of Palestine


### MEDICAL PRACTICE IN PALESTINE.

BY

H. J. Orr-Ewing, M.D., F.R.C.P.,

*Late Consultant Physician, Government of Palestine.*

---

The first point that impresses the medical man in Palestine is the question of languages. At the last census no less than fifteen different mother tongues had to be put down for a group of less than 100 patients and staff, and one never could manage to get through a morning in out-patients without using at least six different languages.

In medical work one comes into close touch with doctors from every country in Europe, each with his own ideas of approach to a case. One learns to be a good deal less insular, and to see that French, German and Italian methods are, in many cases, well worth trying. There are also the native-born doctors, Arab, Armenian and Jew, trained chiefly in the large medical school in Beyrouth. One can observe here the impact of western ideas upon races with cultures of their own, which, however, have been largely stationary for some hundreds of years.

Immediately after the War the country was still under a military or quasi-military régime, the people were recovering but slowly from the aftermath of starvation and deficiency diseases, and British, French, Italian and other voluntary medical organizations were just recommencing those activities which the War had suspended. Roads were bad and transport difficult and very expensive. The American Zionist medical organization, which had been doing first-class work since 1918, was progressing, but had not realized its later stages of efficiency, and the Jews had not yet started to come in in any large numbers. Now roads are first-class to all main towns, transport is ridiculously cheap, and the people are healthier, stronger and better off than ever before. Epidemic disease is energetically combated by an efficient, well-staffed and well-equipped Department of Health ; the French and Italian health organizations are subsidized by their governments for political ends ; there is a German semi-religious organization; and in addition to these are the British Mission hospitals, the Zionist hospitals, and Government hospitals, maternity and welfare centres, and schools of nursing. So that it is a very different medical world from what it was in 1919.

But only a fool would attempt to generalize over even such a little land as Palestine, for in an area no bigger than that of Wales all extremes of climate are to be found, from the breezy uplands of Judæa to the tropical heat of the Jordan valley, and from the foothills and mountain slopes of Hermon to the steamy maritime plain. Nowhere else in the world can such great diversities in temperature and climate be found in such a small space. Even the rainfall varies greatly, for while Jaffa and the foothills may in a good year get 36 inches, Jericho, only fifty miles away, is passing rich on 12 inches. Strictly speaking, Palestine is not tropical in climate (except for certain areas), and does not get the purely tropical diseases to any great extent. But the diseases of the sub-tropical zones are nearly all seen, and also practically all the conditions which fill the majority of the hospital beds at home. For example, in winter a children's ward in Jerusalem (and it can be very cold at 2,600 ft. !) would probably show a majority of broncho-pneumonias, while in summer it would be filled with every variety of infantile diarrhœa from the fulminating cholera nostras to the marasmic child with lienteric stools.

There is, however, no doubt that some diseases are much less common than formerly. Take, for example, **Malaria.** This was the scourge of Palestine ; it still is in certain areas, but in Jerusalem and district the energetic measures adopted by the Government have almost wiped it out. Such measures are the anti-mosquito campaign, compulsory notification and vigorous treatment. Before the War, when Professor Muhlens of Hamburg was investigating the incidence of the disease in hospital patients, it was arranged that blood-films should be taken at the Out-patient Department from every patient presenting with fever. In the course of one year among 2,500 patients whose blood was so examined some 1,200 were found to have malarial parasites in the films, which gives some idea of the frequency of the infection. But during the whole seven years 1927–1933 I saw only two patients who had acquired fresh malarial infections in Jerusalem areas, and both of these had probably been infected in trips to Jericho. Cases still come, of course, who have been infected elsewhere, for parts of Galilee and the Esdraelon plain are still heavily infected, and the infection is very difficult to eradicate.

It is much worse in wet years, which invariably mean greatly increased work for the public health authorities. Yet even in the worst places much has been done by drainage and the use of oil. Beisan (the ancient Bethshan) was one of the worst spots in Palestine with a spleen rate of 40 per cent. I remember Sir W. Willcocks, the engineer, telling me he thought the people there in 1912 were the sickliest he had ever seen. He was conducting an investigation into possible irrigation schemes, which he abandoned on account of the danger from the spread of malignant tertian malaria. Some years ago the Government applied to the Rockefeller Institute for a grant to help the fight against malaria at this spot, but in the meantime the Public Works Department had carried out a drainage scheme for agricultural purposes, and the grant was refused because the spleen rate had by then fallen to less than 2 per cent. Malignant malaria is, however, still a menace in some parts; but benign tertian malaria is much less in amount, and, in fact, now in Jerusalem one is more likely to miss it through not expecting it than, as formerly, to err by expecting it in every febrile case.

**Plague,** of course, has frequently attacked Palestine in times past. The emerods of 1 Samuel v. were probably plague buboes associated with a plague of mice (were these rats, or were they a plague of field-mice such as has ravaged the land of recent years ?) The Philistine plain has always been a place where invading armies have been decimated by disease, as were Sennacherib's and Napoleon's. But of late years only one (easily controlled) epidemic has occurred. The black rat is the indigenous member of his race, but the sanitary service has so far proved too much for the *bacillus pestis.*

The country is from its position peculiarly vulnerable to such diseases as **Cholera** (which in days not long ago depopulated the towns), because it is only just off the track of the great Hadj or pilgrimage to Mecca, and pilgrimages are fruitful sources of cholera. But stringent quarantine regulations and examinations have so far during the present régime kept Palestine clear of this dread visitant, although Irak has suffered. **Typhoid** is common, but very atypical in manifestation. One learns to suspect it in any febrile case lasting more than five days and resistant to quinine. Paratyphoid B. is also not rare. The mortality is low, and although hæmorrhage is quite common, perforation is so rare that I have never seen a case. Bacillary **Dysentery** is quite common, both Flexner and Shiga strains being found. The former is the more frequent, but the latter the more virulent. Amœbic dysentery is seen much less often, though it does occur; but liver abscess is rare.

Other diseases which are very common in neighbouring countries, such as Egypt, affect Palestine very little owing to the absence of water. **Ankylostomiasis,** for example, is almost unknown. **Bilharzia,** however, was introduced into the swampy district north of Jaffa by members of the Egyptian labour corps during the War, and finding the intermediate host, the freshwater snail, proceeded to establish itself: there are now a number of cases reported from the area. A schoolboy with hæmaturia was found to have abundant ova in the urine, and this led to the examination of the urine of all the boys in the school.

It was found that some 20 per cent. were infected, although nearly all were without any symptoms. But the disease can never make headway in the dry upland districts.

Cutaneous **Leishmaniasis,** known locally as "Jericho boil," is becoming increasingly prevalent. It is common in Syria, where it is called "Aleppo button," affecting 70 per cent. of the inhabitants of that city. Until recently it was in Palestine confined to Jericho and the Jordan valley, where the sand-flies are heavily infected, and moreover to persons who stayed in the valley during the summer months. Visitors up to the end of March escaped, for the sand-fly does not become active until later. As no one willingly stayed down in the cauldron that the valley becomes in summer, the disease was by no means widespread. But now cases are reported from widely-separated places in the hills and plains. The probable explanation is that nowadays with motor transport going through the valley at all seasons on the way to Transjordania many people remain there long enough to be infected, and then go their way to their home towns and infect the sand-flies there. The mode of transmission of the disease by sand-flies was worked out in Jerusalem by Adler, and forms a strong link in the chain of evidence he is accumulating against that insect as the carrier of the other form of Leishmaniasis, Kala-azar. Curiously enough this is almost unknown in Palestine, although I have seen a case; with the presumed host present in large numbers and the cutaneous parasite widespread one would have expected at least a modicum of cases. The treatment of "Jericho boil" is not altogether satisfactory, and much scarring usually ensues. The new drug *orisol* is spoken of very highly, but I have had no personal experience of its use.

**Dengue** fever, " Father of Knees " as the Arabs call it from the severe pains in the legs, is not very often met with in Palestine, thanks to the intensive anti-mosquito measures, but in Syria it rages at times, attacking up to 70 per cent. of the population. It has to be distinguished from the short, sharp, very weakening **Papataci** or **Sand-fly Fever. Typhus** occurs at intervals, chiefly among the poorer Jewish immigrants, but lice are not so prevalent amongst the Arabs as are fleas and bugs. Among these, therefore, it is not often seen, nor is relapsing fever. **Mediterranean Fever** occurs in the plains, but the goats are not heavily infected.

Enough has been said to show that genuine tropical diseases are not very common, and a few remarks must be made about other diseases better known at home. In Palestine, as here, **Pneumonia** is still " Captain of the men of death," but the most dreaded of all diseases is **Tuberculosis.** It is especially severe amongst the Yeminite Jews, recent arrivals from Aden and the neighbourhood, where the scourge does not occur. At any rate, in the damper and colder climate of Palestine they speedily fall victims to pulmonary tuberculosis. The Bedouin suffer badly from bone and joint tuberculosis, as also do the inhabitants of Nablous (Shechem). One can only suppose that the nomadic lives of the former people and the fanatical outlook of this most bigoted Moslem town have prevented them acquiring that immunity which contact with the disease has conferred on others.

Of the exanthemata **Scarlet Fever**, which never occurs in the tropics, had never been seen before 1926, in which year we had a widespread but mild epidemic. **Measles** is, perhaps, the most fatal of all diseases to the child population, the death-rate in some epidemics being appalling. It is much more fatal amongst the Arabs than amongst the Jews. 1,000 out of 3,000 children are said to have died in one town alone in Transjordania in the course of a single epidemic. And I remember having seen a whole family of three children lying dead in one room. The cause of death is much more often gastro-intestinal than pulmonary complications, except in the middle of winter. **Small-pox** is greatly dreaded, and the appearance of a case is the signal for a wave of vaccinations by the thousand. It was introduced from Syria in 1923, and sporadic cases occur at intervals. But for some years there has been none, and the town population, at least, is kept well vaccinated.

**Syphilis** is extremely common: in some villages 90 per cent. of the inhabitants give positive Wassermann or Kahn tests. It is definitely to be traced to the conscription which the Turks enforced when they first began to impose law and order on the most turbulent districts; the men were conscripted and taken off to Constantinople and so on, where they became infected, and on return infected their wives. Everyone uses common drinking cups in the villages, and soon everyone was infected. Rashes and primary lip chancres are seen, but far the most common signs are mucous patches in the mouth and throat. This state of affairs is very common around Jerusalem (where nothing would induce me to drink coffee with the villagers). It was worse after the War, due to further infections during the War. Special syphilis clinics have been instituted by the Government. Genital chancres are rare, and are looked on as a disgrace and called " European boil," while the usual form is called " Bejl " (origin unknown), and not in any way associated with venereal disease. Similar conditions exist 600 miles away on the Euphrates.

**Rabies** is very rare now, for the Government Antirabic Institute treats free and immediately all persons bitten or scratched by suspected animals. The last cases were in Samaria, where a mad wolf (itself now a rarity) ran down the main street of a village and bit four persons, two in the face—always the most fatal bite—and these two died. They presented all the classical features of convulsions, hydrophobia, etc. On the other hand, a man who was badly bitten by a mad wolf in 1931 escaped infection, and a month after his treatment, upon his return to his post as shepherd in the Jordan valley, was again bitten, this time by the rabid mate of the first aggressor. Although this sounds incredible it is perfectly true. The jackal forms a reservoir for rabies, and domestic dogs are now given prophylactic inoculation; I lost a valuable Great Dane when it was inoculated.

I am not competent to deal with the very common eye diseases, but there are many intriguing problems which cry out for solution. Why is appendicitis very much more common now than it was? Why does one *never* see intussusception? Why, although peptic ulcer is one of the common conditions, and hæmatemesis by no means rare, is perforated ulcer so rare that I only saw one case in twelve years? Is it really a fact that the whole of the increase in cancer is only "apparent" and due to better diagnosis? Whatever the answers to these, I think I may claim that medical practice in Palestine is interesting, varied and—I at least found it—very engrossing.

